# Chylothorax After Thoracic Surgery: How We Manage It

**DOI:** 10.1111/1759-7714.70036

**Published:** 2025-03-06

**Authors:** Alberto Busetto, Giorgio Cannone, Luigi Lione, Alessandro Bonis, Vincenzo Verzeletti, Michele Battistel, Alessandro Rebusso, Samuele Nicotra, Andrea Dell'Amore, Federico Rea

**Affiliations:** ^1^ Thoracic Surgery Unit, Department of Cardiac, Thoracic, Vascular Sciences and Public Health University of Padova Padova Italy; ^2^ University Radiology, Department of Medicine University Hospital of Padova Padova Italy

**Keywords:** chylothorax, indocyanine green fluorescence, lymphangiography, lymphatic embolization, thoracic surgery

## Abstract

Chylothorax is a rare but insidious condition, characterized by the accumulation of chyle in the pleural space, which is particularly common after cardiothoracic surgeries. It presents significant challenges in both diagnosis and treatment. In this technical report, we present our experience in managing four cases of postsurgical chylothorax, each one treated with a different approach. The first and second cases were successfully managed with Lipiodol lymphangiography, which allowed for the visualization and occlusion of the injured lymphatic duct, leading to the resolution of the chylothorax. The third case involved thoracic duct embolization, a procedure that resulted in the closure of the duct responsible for the chylous effusion. The last case involved a patient who developed left‐sided chylothorax following a pulmonary resection. The patient experienced chylous leakage early in the postoperative period and underwent a revision thoracoscopy for hemostasis and thoracic duct ligation. During the procedure, indocyanine green (ICG) fluorescence was used to effectively identify and ligate the injured chylous duct. This case series highlights the variety of therapeutic strategies available for the management of chylothorax, emphasizing the importance of a structured, stepwise approach tailored to the specific needs of each patient.

## Graphical abstract

Chylothorax After Thoracic Surgery: How We Manage It. We present our experience in managing different presentations of post‐surgical chylothorax, treated either conservatively or surgically. Conservative treatment: (1) Somatostatin (2) Parenteral nutrition. Interventional Radiology treatment: (1) Lipiodol lymphangiography (2) Thoracic duct embolization. Surgical treatment: (1) Duct ligation (right‐sided/left‐sided) (2) ICG guided.

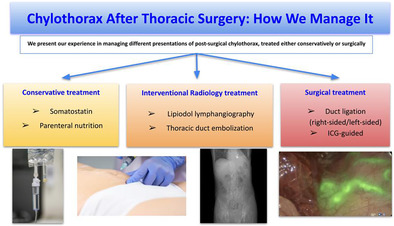

Chylothorax is a pathological condition characterized by the accumulation of chyle in the pleural cavity. The most common cause of chylothorax is trauma, particularly following thoracic procedures, including esophageal surgery and mediastinal lymphadenectomies. Nontraumatic causes of chylothorax include malignancies, granulomatous diseases, and infectious conditions. Clinical manifestations of chylothorax depend on the volume and rate of chyle accumulation in the pleural space. Initial treatment is conservative, focusing on reducing chyle production with a lipid‐free diet or somatostatin administration. Persistent leakage may require total parenteral nutrition. High‐output, refractory chylothorax or failure of conservative approaches often necessitates more invasive management, such as lymphangiography, which can be used both as a diagnostic and a therapeutic tool, as it allows for the identification of the leakage site and embolization of the injured lymphatic vessel. Surgical ligation of the duct is reserved for cases that do not respond or for relapsing cases, and it involves the ligation of the thoracic duct, usually from the right hemithorax, with a success rate of about 90%. Mass ligation of all the tissue between the aorta, spine, esophagus, and pericardium or talc pleurodesis may be performed in cases where it is not possible to properly identify the leak site [1, 2, 3, 4]. The use of indocyanine green (ICG) fluorescence during thoracoscopy in such tricky conditions may be of great help in successfully identifying and treating the chylous leakage site [5, 6].

In this technical report, we present our diagnostic‐therapeutic algorithm for the management of four different cases of postsurgical chylothorax that occurred in our Thoracic Surgery Unit, including technical notes and challenges.

The first case we present is about a 58‐year‐old male affected by pulmonary adenocarcinoma in the left lower lobe. The patient underwent left lower lobectomy and radical lymphadenectomy through posterolateral thoracotomy. Final histologic examination deposed for a pT4N0 stage lung adenocarcinoma. The patient was discharged on postoperative day 4 with no complications. A chest X‐ray scan after 20 days postsurgery revealed a moderate amount of left pleural effusion. As a result, we placed a chest drain with chylous fluid drainage. The patient was placed on total enteral nutrition and somatostatin administration. Due to persistent serous leakage from the chest tube, our Interventional Radiologist performed lymphangiography through bilateral inguinal lymph node access (Lipidiol 10 mL for each side). The following day, a chest CT scan revealed contrast agent accumulation in the left pleural cavity (Figure 1). Two days after the lymphangiography, the patient underwent successful percutaneous thoracic duct embolization (Figure 2). The second case involves a 71‐year‐old woman diagnosed with adenocarcinoma of the right upper lung lobe. She underwent a right upper lobectomy associated with hilar and mediastinal lymphadenectomy through a right triportal VATS approach. On the third postoperative day, chylous leakage from the chest tube was observed. After initial conservative management, a lymphangiography was performed on the seventh postoperative day, with the injection of approximately 8 cc of Lipiodol into each inguinal lymph node. Immediately after the procedure, the fluid loss from the chest tube ceased. The third case involves a 34‐year‐old man with non‐Hodgkin lymphoma diagnosed via thoracoscopic biopsy in November 2023. In June 2024, during follow‐up, an echocardiogram detected a pericardial effusion with minimal hemodynamic instability. Persistent chylous leakage of around 100 mL from the pericardial thoracostomy was noted daily. As a result, in August, interventional radiologists performed a bilateral percutaneous lymphangiography with Lipiodol injection through the inguinal lymph nodes. This first procedure was unsuccessful, and the chylous drainage persisted at similar volumes. A second procedure took place 1 week later, with the same contrast agent dosing. One day after the second procedure, the patient underwent a chest CT scan, showing evidence of the tracer at the level of the abdominal‐thoracic lymphatic system. Subsequently, Radiologists successfully performed fluoroscopic cannulation of the tracked lymphatic vessels and their embolization with cyanoacrylate. The last case presented is about a 54‐year‐old female with a pulmonary PET‐positive lesion in the left upper lobe. The patient underwent triportal thoracoscopic wedge resection of the left upper lobe with hilar‐mediastinal lymphadenectomy. On postoperative day 1, the patient developed chylous drainage from the pleural drain. On postoperative day 7, we noticed active bleeding from the drainage with a mean of 100 mL/h of hematic output, and we decided to return the patient to the operating room for evacuation of hemothorax and hemostasis. Concurrently, 3 mg of ICG diluted in 5 mL of saline solution was injected bilaterally into the inguinal lymph nodes by our Interventional Radiologists. With the ICG filter in the thoracoscopic camera, lymphatic vessels on the left side were clearly visualized, and the leakage point was precisely identified at the level of the previous carinal lymphadenectomy. The leak was successfully sealed with metal clips (Figure 3) and with the application of nebulized cyanoacrylate for further tissue sealing.

**FIGURE 1:**
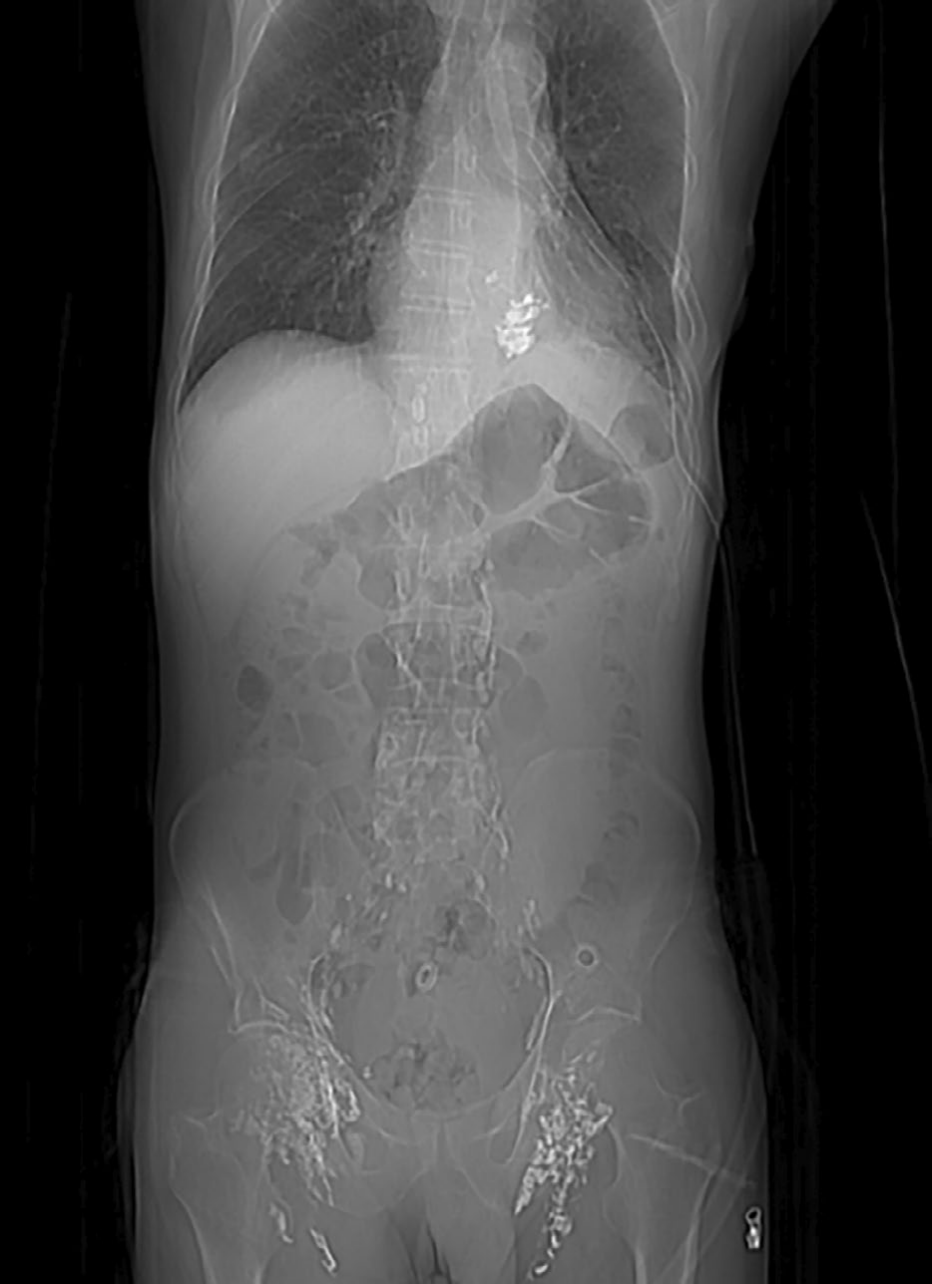
Evidence of contrast agent accumulation in the left pleural cavity and along the lymphatic chain from the inguinal lymph nodes, as detected through a chest CT scan.

**FIGURE 2:**
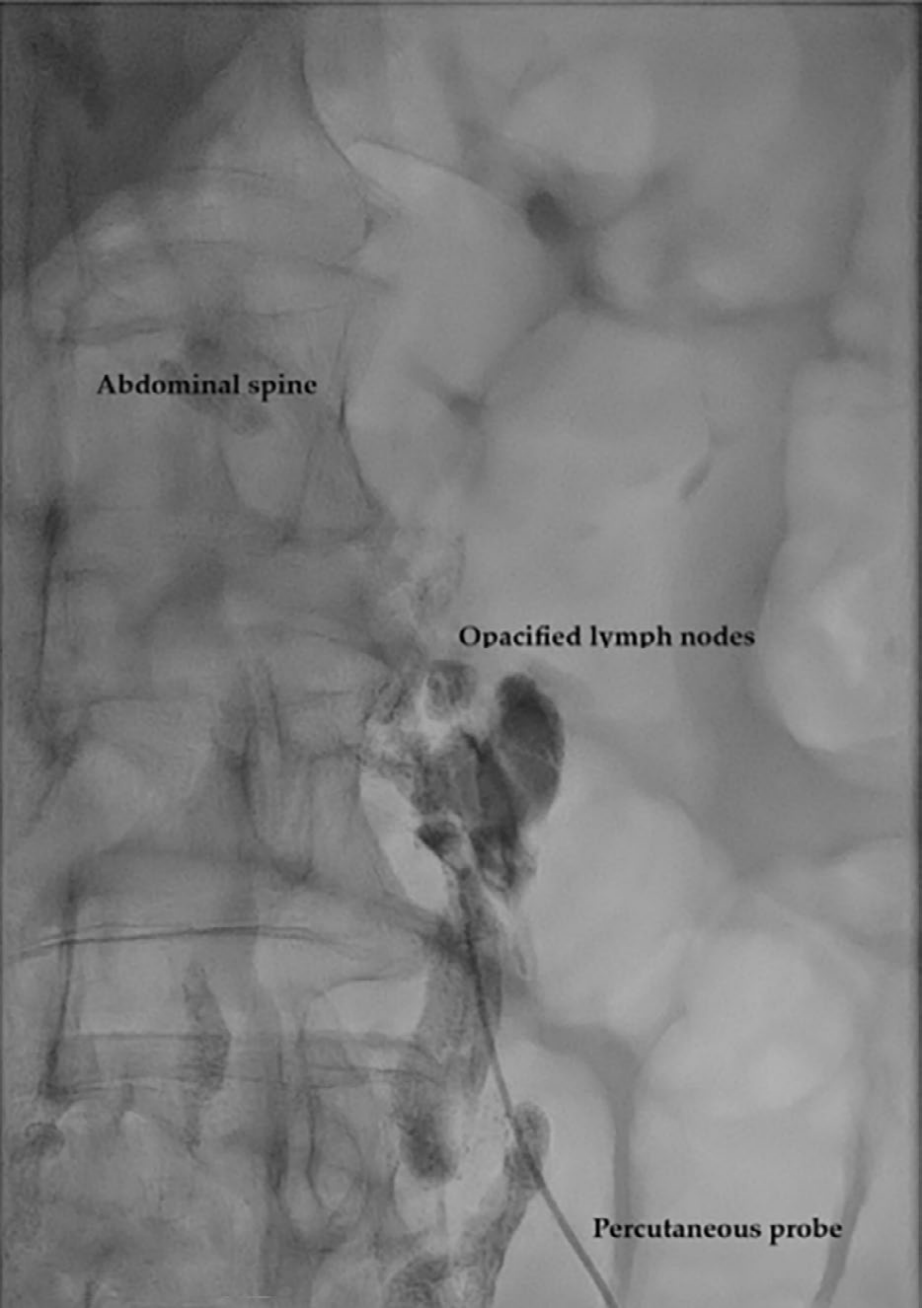
Attempt to embolize the thoracic duct while ascending the opacified abdominal lymphatic system through percutaneous access.

**FIGURE 3:**
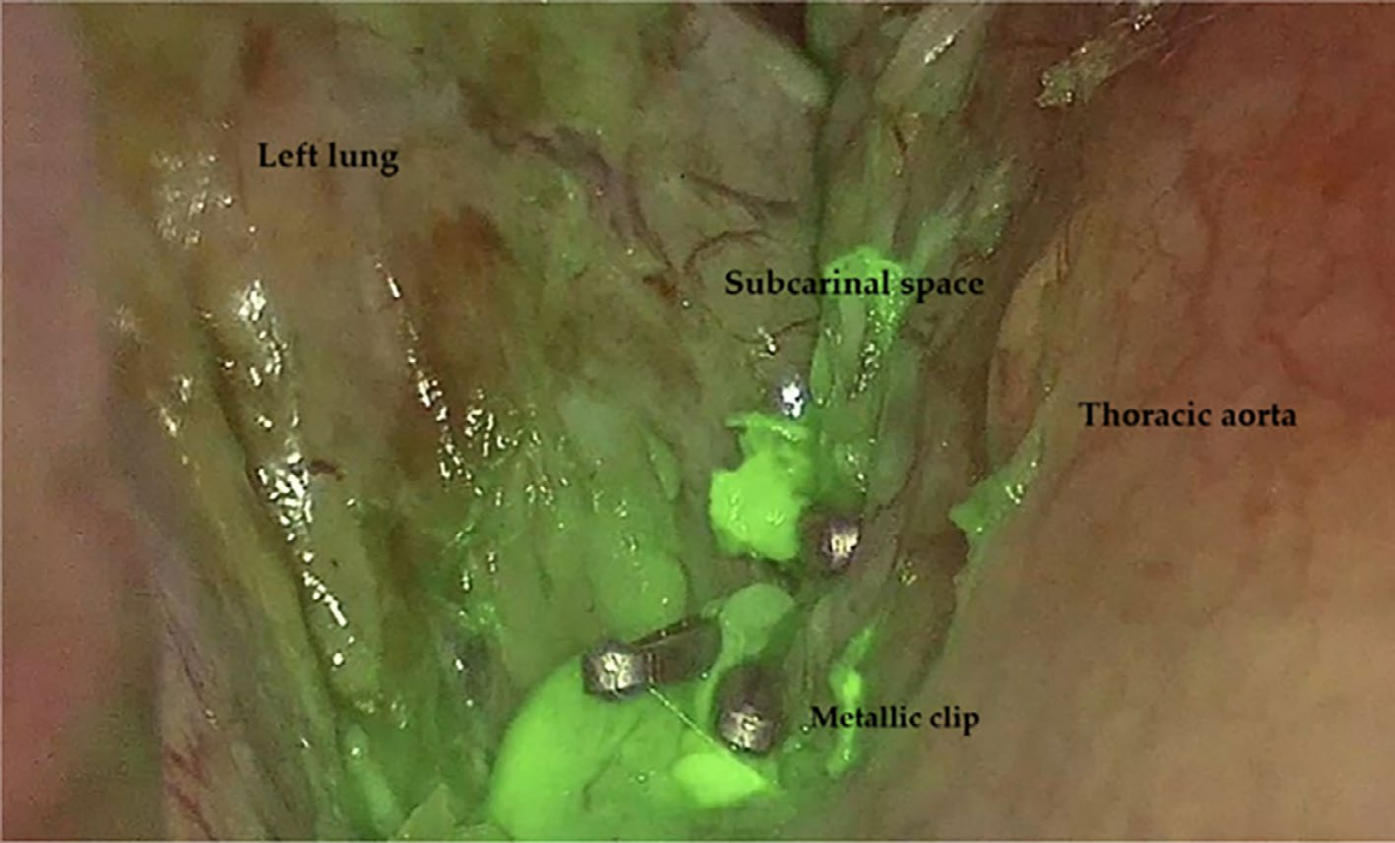
ICG‐fluorescence highlighting the leakage site in the left subcarinal space, along with the metal clips used to close it.

In this report, we presented diverse types of occurrences of chylothorax and different management strategies. Variations in thoracic anatomy and the presence of numerous accessory vessels complicate the already difficult identification of the thoracic duct and its affluents during surgery. Conservative management, including dietary modification and somatostatin, is effective in some cases; however, this approach fails in approximately 30%–50% of cases [4], particularly when the chylous output exceeds 1 L per day or persists for more than 2 weeks. The role of somatostatin and its analogs has been valuable in many patients, but it does not achieve complete resolution in a significant number of cases, further supporting the need for early escalation to procedural interventions [7]. An interventional strategy, whether surgical or percutaneous, should be considered in patients with a persistently high volume of chylous output or those with a prolonged leak before complications such as malnutrition occur [3]. Invasive diagnosis and treatment include percutaneous lymphatic duct embolization and surgical approaches. In our center, the first step for embolization involves bilateral inguinal lymphangiography using Lipiodol contrast material. This is performed to visualize the lymphatic tree and all its anatomical variants. A direct thoracic/abdomen CT scan is performed within 24 h from the procedure, which allows us to identify the possible leakage of the contrast agent into the pleural cavity (Figure 1). Once the certainty of the Lipiodol leakage is assessed radiologically, the Interventional Radiologists proceed with the lymphatic duct embolization. Access to the lymphatic system is then established either by catheterizing the major lymphatic ducts, such as the thoracic duct, or by inserting small needles into the surrounding tissue. Once access is achieved, cyanoacrylate associated with oils is injected to embolize the lymphatic system [8, 9].

Surgical intervention is reserved for relapsing cases, for conditions of elevated lymphatic output, or for other conditions where lymphangiography is not feasible. The most widely used approach is duct ligation from the right hemithorax. In case the leakage is properly visualized, the ligation may be targeted; otherwise, the tissue between the aorta, spine, and esophagus can be ligated. Indocyanine‐green fluorescence contrast agent can be a great help in identifying the lymphatic leak. ICG fluorescence is widely used in various surgical fields for intraoperative localization of lesions and real‐time visualization of segmental anatomy. Our diagnostic‐therapeutic flowchart is summarized in the image below (Figure 4).

**FIGURE 4:**
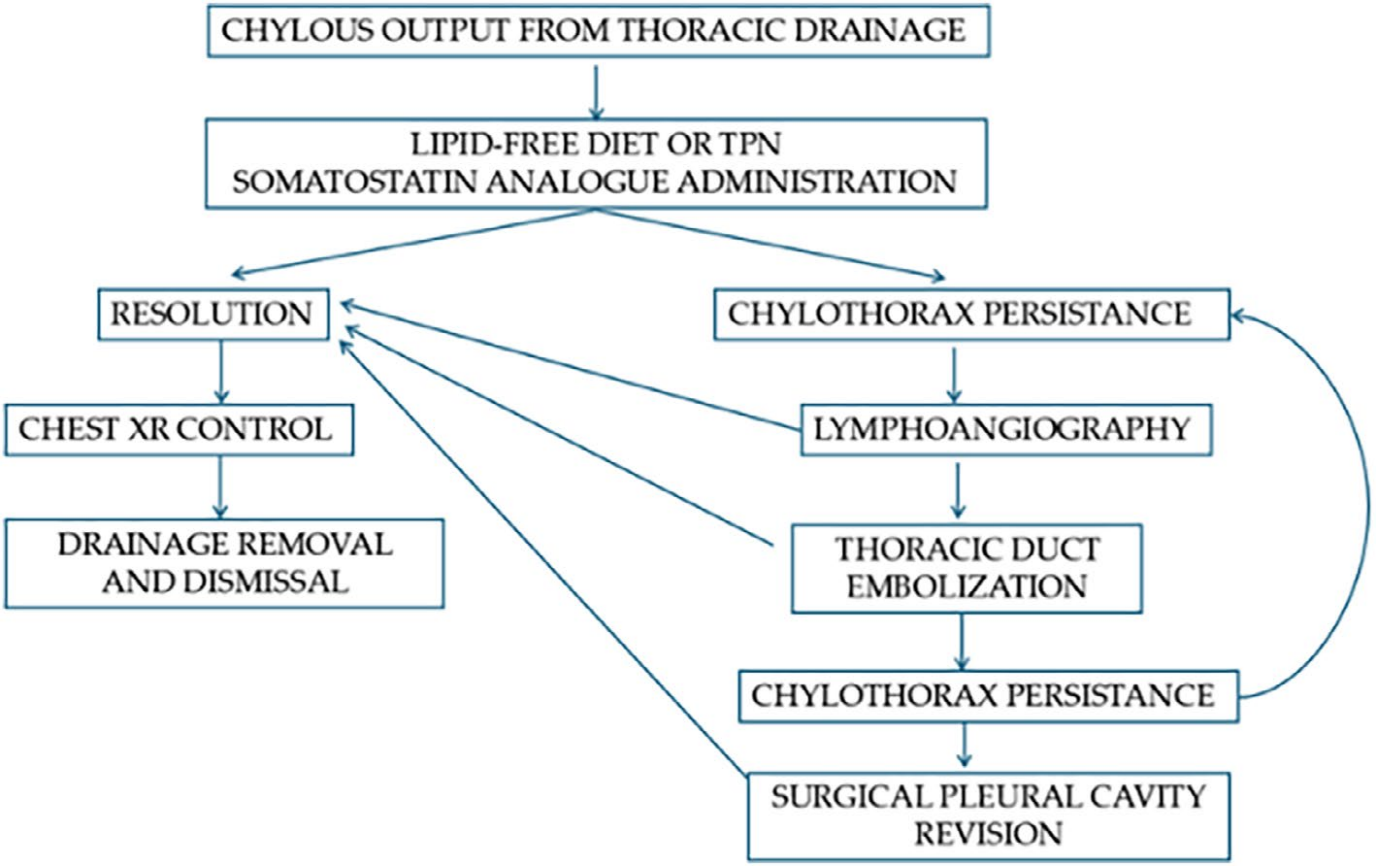
Diagnostic and therapeutic flowchart used for the management of chylothorax in our Thoracic Surgery Unit.

In conclusion, this technical report demonstrates the diverse presentations and therapeutic approaches for managing postsurgical chylothorax. The variability in both anatomical patterns and the extent of chylous leakage necessitates a tailored, stepwise approach for each patient. Techniques such as lymphangiography, thoracic duct embolization, and surgical ligation have proven successful in resolving chylous effusions when conservative measures fail. Additionally, the use of ICG fluorescence during thoracoscopy has shown promise in precisely identifying and ligating the leaking lymphatic vessels, particularly in challenging cases where traditional methods may fall short.

## Author Contributions

Conceptualization: Alberto Busetto, and Luigi Lione, Methodology: Giorgio Cannone and Michele Battistel, Software: Alessandro Bonis, and Vincenzo Verzeletti, Validation: Alberto Busetto, Giorgio Cannone, and Luigi Lione. Formal analysis: Samuele Nicotra, and Andrea Dell'Amore. Investigation: Alberto Busetto. Data curation: Samuele Nicotra and Alessandro Rebusso Writing – original draft preparation: Alberto Busetto and Luigi Lione. Writing – review and editing: Alessandro Bonis, Giorgio Cannone, and Vincenzo Verzeletti. Visualization: Giorgio Cannone. Supervision: Samuele Nicotra, Alessandro Rebusso, Andrea Dell'Amore, and Federico Rea. Project administration: Giorgio Cannone. All authors have read and agreed to the published version of the manuscript.

## Consent

All patients involved in the study provided written informed consent to participate.

## Conflicts of Interest

The authors declare no conflicts of interest.

## Data Availability

The data that support the findings of this study are available on request from the corresponding author. The data are not publicly available due to privacy.
